# Animal Models of Systemic Sclerosis: Using Nailfold Capillaroscopy as a Potential Tool to Evaluate Microcirculation and Microangiopathy: A Narrative Review

**DOI:** 10.3390/life12050703

**Published:** 2022-05-08

**Authors:** Angélica Mandujano, Melissa Golubov

**Affiliations:** 1 Departamento de Atención a la Salud, Universidad Autónoma Metropolitana-Xochimilco, Mexico City 04960, Mexico; 2 Facultad de Medicina, Universidad Nacional Autónoma de México, Mexico City 04510, Mexico; mc20gomm2292@facmed.unam.mx

**Keywords:** systemic sclerosis, Raynaud’s phenomenon, nailfold capillaroscopy, animal models, fibrosis, vasculopathy

## Abstract

Systemic sclerosis (SSc) is an autoimmune disease with three pathogenic hallmarks, i.e., inflammation, vasculopathy, and fibrosis. A wide plethora of animal models have been developed to address the complex pathophysiology and for the development of possible anti-fibrotic treatments. However, no current model comprises all three pathological mechanisms of the disease. To highlight the lack of a complete model, a review of some of the most widely used animal models for SSc was performed. In addition, to date, no model has accomplished the recreation of primary or secondary Raynaud’s phenomenon, a key feature in SSc. In humans, nailfold capillaroscopy (NFC) has been used to evaluate secondary Raynaud’s phenomenon and microvasculature changes in SSc. Being a non-invasive technique, it is widely used both in clinical studies and as a tool for clinical evaluation. Because of this, its potential use in animal models has been neglected. We evaluated NFC in guinea pigs to investigate the possibility of applying this technique to study microcirculation in the nailfold of animal models and in the future, development of an animal model for Raynaud’s phenomenon. The applications are not only to elucidate the pathophysiological mechanisms of vasculopathy but can also be used in the development of novel treatment options.

## 1. Introduction

Systemic sclerosis (SSc) is a chronic autoimmune disease with a wide range of clinical manifestations. The classic pathogenic triad includes the activation of innate and adaptive immune responses resulting in inflammation, vasculopathy, and fibrosis [1,2]. From a pathophysiological point of view, it is not completely clear whether tissue damage and cell activation are initiated as a result of altered fibroblast activation or due to endothelial cell damage, as both have been implicated.

Vascular features are of the utmost importance, not only because they are almost always present, but also because they are clues to suspect diagnosis in the early course of the disease, long before the appearance of non-vascular manifestations [3,4]. Although vascular damage and endothelial cell apoptosis present quite early [2,5] and there is a close relationship between vasculopathy and fibrosis, the critical events that lead to persistent and uncontrolled fibroblast activation and deposition of extracellular matrix proteins have not been fully elucidated. In this sense, it is necessary to elucidate the relationship between alteration of endothelial cells, fibroblasts, and immune activation in order to disrupt the chain of damage that leads to development of the disease. Despite this, it is clear that vascular manifestations play a central role in the disease. However, there is still little knowledge about the pathophysiology and crucial factors that induce endothelial damage and about the central targets that could induce vascular damage, which could be later used to improve current therapeutic strategies.

Within the vascular manifestations, Raynaud’s phenomenon (RP) is present in ~95% of patients [3,4]. It is a clinical feature that may guide diagnosis, especially if it is accompanied with nailfold capillaroscopy abnormalities. Other clinical features associated with persistent digital ischemia include digital pitting scars, digital ulcers (in around 50% of SSc patients at some point of the disease), and gangrene, all of which are an import cause of morbidity in these patients [3,4]. Furthermore, using videomicroscopy, sublingual abnormalities have been associated with vasculopathy. In addition, some capillary abnormalities correlate with nailfold capillary findings [6]. Unlike other clinical manifestations such as pulmonary arterial hypertension (PAH), interstitial lung disease or scleroderma renal crisis [7], RP and nailfold capillaroscopy abnormalities have not been correlated with specific autoantibodies.

SSc has a very complex pathophysiology in which the interaction of several environmental [8,9,10,11], hereditary [12,13,14,15], and epigenetic modifications [11,16,17,18,19,20] have been involved. Although there are many mediators that have been shown to be involved in the disease, it has not been sufficient for the development of effective disease-modifying therapies, to the point that it remains one of the most fatal rheumatologic diseases [21]. This is due largely to the lack of a single animal model that represents the wide range of manifestations of human SSc. Current animal models recapitulate one or more aspects of SSc [22], but never all, and additionally lack the variety of vascular features that occur in SSc beyond PAH. In fact, there is no appropriate animal model for RP [23], which has limited the study of primary and secondary RP, a very common manifestation, in the context of SSc and other autoimmune diseases. The aim of this narrative review is to highlight the lack of an animal model for RP that resembles SSc vascular manifestations and to propose nailfold capillaroscopy (NFC) as a feasible tool for the study of microcirculation in animal models of SSc as an additional method for the evaluation of vasculopathy. Application of NFC to animal models could potentially contribute to the development of an RP model.

## 2. Methods

We performed a literature search in PubMed and MEDLINE complete/EBSCO databases. Articles chosen were limited to those written in English and there was no date limit. We used key words such as systemic sclerosis, scleroderma, animal models, fibrosis, vasculopathy RP, and nailfold capillaroscopy. Review articles about animal models of systemic sclerosis, original articles on animal models development, and clinical studies related to targets described in animal models were included.

## 3. Results

### 3.1. Animal Models Induced by Administration of Exogenous Agents

There are animal models that use fibrosis-inducing agents such as subcutaneous bleomycin for skin fibrosis [23] or intratracheal administration for pulmonary fibrosis [24,25]. The bleomycin-induced model is one of the most widely used models to study both skin fibrosis [26,27,28] and pulmonary fibrosis [29,30,31]. One of the most striking features in this model is that fibrosis resolves over time with discontinuation of treatment. Although it is an appropriate model for skin fibrosis (and lung fibrosis), studies with this model have not reported the characteristic vasculopathy of SSc. Only Yamamoto et al. found vascular wall thickening in the deep dermis [32]. A different model described by Servettaz and collaborators (2009) was induced by generation of reactive oxygen species (ROS) with prooxidative agents (hydroxyl radicals, hypochlorous acid, peroxynitrites, and superoxide anions). Interestingly, they found that either hypochlorite or hydroxyl radicals can induce systemic disease, cutaneous and lung fibrosis, and anti-topoisomerase I autoantibodies; while those treated with peroxynitrites showed skin fibrosis and anti-centromere protein B antibodies (CENP-B). Another remarkable finding was a reduction in the lumen diameter and intimal fibrosis with intima-media thickening in small renal arteries in mice treated with either hydroxyl radicals or hypochlorous acid [33]. Skin and lung vasculopathy, as well as lung and skin fibrosis, has been ratified by treating this model with hypochlorous acid [34].

Another model is induced by multiple intraperitoneal injections of vinyl chloride, where activation of microchimeric cells leads to dermal and organ fibrosis [35,36,37].

Exogenous subcutaneous injections of transforming growth factor-β TGF-β, connective tissue growth factor (CTGF), and basic fibroblast growth factor (FGF) induce fibrosis in murine models. TGF-β administration induces only transient fibrosis; however, CTGF and FGF lead to persistent fibrosis. It is noteworthy that multiple injections and the use of a combination of the above factors are required to develop lasting fibrosis [32,36]. These and other models are described in greater depth in Table 1.

### 3.2. Natural or Induced Mutant Models Associated with SSc-like Disease

Tsk1/^+^ is a spontaneous autosomal dominant mutation of the B10.-D2(58N)/Sn mice strain, affecting fibrillin function [38,39]. It is a tandem duplication within the fibrillin-1 gene [39]. Heterozygous Tsk1/^+^ mice develop SSc-like features, with important differences regarding involvement of the lung and skin [32,38,39,40]. Tsk2 is generated by administering ethylnitrosourea, which causes an autosomal dominant mutation in chromosome 1 in the offspring of a 101/h strain mice [32,39]. Long et al. reported that Tsk2/^+^ mice carry a gain-of-function missense point mutation in the procollagen III amino terminal propeptide segment of Col3α1 [41]. Heterozygous mice showed tight skin as seen in TsK1, but with no lung involvement [42].

Of the animal models, the UCD 200 and 206 chicken lines are among the most relevant because they manifest all the hallmarks that most closely resemble human SSc. In the UCD 200 model, endothelial cell apoptosis was identified as an early pathogenic event [5]. A more detailed description of these models is found in Table 1.

### 3.3. Genetically Engineered Mice Models

Genetically modified models are a powerful methodology to investigate the pathogenesis and therapeutic targets of human diseases. For SSc, there is a mice strain overexpressing the fibroblast kinase-deficient type II TGF-β receptor (TβRΙΙΔk-fib mice). This represents a loss-of-function mutation, which paradoxically develops progressive skin fibrosis especially in the lower back, lung, and gut in a proportion of cases [22,32,39,43]. This same strain showed medial thickening and mild PAH; however, following administration of a VEGF inhibitor, it developed more severe PAH [44]. A type I TGF-β receptor transgenic model also showed upregulation of the TGF-β pathway by fibroblast upregulation of the receptor [38,39].

A fibrillin-1 (Fbn1)-target knock-in mutant mice displayed several features of a systemic sclerosis-like disease, including skin fibrosis, immune cell activation, and autoimmunity with antinuclear and anti-topoisomerase I antibodies, but did not develop microvascular changes [22].

Transgenic mice expressing Fra-2 (Fos related antigen 2) show apoptosis of dermal endothelial cells, rarefication of skin capillaries, as well as skin and lung fibrosis, the latter preceded by obliteration of pulmonary arteries and perivascular inflammation [22,45]. Fli-1 (Friend leukemia integration 1) and kruppel-like factor 5 deficient mice, both with a heterozygous deficiency, develop skin fibrosis, obliterative vasculopathy, and autoantibodies [20]. Mice with urokinase-type plasminogen receptor (uPAR) deletion develop skin and lung fibrosis, as well as microvascular endothelial cell apoptosis with reduced dermis and myocardium capillary density [46,47]. Sirt-3 knockout is another model of PAH and systemic fibrosis [22].

A transgenic endothelin-1 mouse model was generated by transferring the natural promoter into germinal mice; this transgene was expressed mainly in the brain, lung, and kidney. Animals showed interstitial fibrosis of the kidneys and glomerulosclerosis but surprisingly, despite its powerful vasoconstrictor effect, systemic hypertension was absent. This renal fibrosis caused a decrease in the glomerular filtration rate that led to fatal kidney disease. However, instead of developing hypertension, the vascular transgene expression was related to an increase in the media/lumen ratio of intrarenal arteries [48]. In addition, in a subsequent study, chronic expression of endothelin-1 was related to progressive pulmonary fibrosis associated with inflammatory cell infiltration, but interestingly, did not result in significant pulmonary hypertension [49]. In a similar gain-of-function approach, the effects of increased PDGFRα activation limited to cells that normally express the receptor was investigated. Increased PDGFRα signaling led to increased extracellular matrix deposition and hyperplasia of connective tissue with a phenotype similar to SSc. Skin tightening was related to excessive connective tissue deposition and fibrosis of muscle, heart, kidney, and intestine with infiltrates of activated fibroblasts [50]. In addition, in an SSc animal model induced by hypochlorous acid, the phosphorylation of PDGFRβ, which reflects receptor activation, was higher in fibrotic skin [51].

In order to address the possible role of caveolin-1 in caveolae function and signal transduction, Drab and coworkers developed a caveolin-1 knockout mouse (Cav-1). This knockout mouse resulted in the absence of caveolae in endothelial and epithelial cells of lungs and all tissues evaluated including the heart, kidney, and adipose tissue. Interestingly, it was found that this model developed marked pulmonary fibrosis [52]. Increased accumulation of dermal collagen fibers extending to the deeper dermis and around skin appendages was later reported [53]. Studies on pulmonary fibroblasts have shown that TGF-β negatively regulates the expression of caveolin-1. On the contrary, the use of an adenovirus to overexpress caveolin-1 significantly decreased ECM production induced by TGF-β. Disruption of TGF-β signaling by caveolin-1 has been explained by the suppression of phosphorylation of smad-2 in pulmonary fibroblasts [54] and smad-3 in dermal fibroblasts [55]. In cell cultures, the use of caveolin-1 has shown that by decreasing the phosphorylation of Smad-2, it disrupts its interaction with Smad-4 and prevents nuclear translocation of Smad-2. Inhibition of Smad-2 is due to the interaction of the caveolin-1 scaffolding domain with TβRI, which leads to inhibition of the receptor’s kinase activity, thus causing suppression of TGF-β signaling [56].

The MRL/lpr strain is used as a model of systemic autoimmunity, but an MRL/lpr deficient for IFN-γ mice has also been proposed as as a model of SSc. This model lacks the IFN-γ receptor (MRL/lprγR−/−) and with the absence of the IFN-γ antifibrotic response, results in dermal and organ fibrosis and vascular damage [57].

Another interesting model of severe fibrosis in the rat lung is due to the activation of the TGF-β pathway by the Ad-TGFβ1^223/225^ adenovirus construct; however this is only a pulmonary fibrosis model [58].

### 3.4. Animal Models with Manipulation of the Immune System

Chronic sclerodermatous graft-versus-host disease (GVHD) resembles fibrotic changes of SSc. There are a couple of models that use this approach. Hamilton and coworkers produced an animal model for both acute and chronic forms of GVHD. For this purpose, they administered a lethal dose of γ radiation in C57BL/6J mice (recipients), and later these mice received bone marrow and spleen cells from LP/J (donors) mice. Those with chronic GVHD develop fibrotic changes in the skin, primaily in the ears, which includes fibrotic bands that restricted extremity mobility. Some of them presented diffuse skin thickening that led to loss of all of their fur. Histologically, the latter was characterized by dermal fibrosis [59]. Another model was described by Claman et al. in 1985 [60]. In this model, spleen and bone marrow cells from B10.D2 mice were transplanted into sub-lethally γ-irradiated BALB/c mice that differed in minor histocompatibility antigens [61]. Skin and lung fibrosis was observed in transplanted animals compared to animal controls. The last model was developed by Ruzek and coworkers. It was induced by spleen cell transplantation from B10.D2 mice to BALB/c recombination-activating gene 2 (RAG-2)-deficient targeted mice, therefore lacking mature T and B cells. They found dermal thickening primarily in extremities and to a lesser degree in the back and abdominal skin. In this model, there was a significant progressive decrease in the lumen diameter of the skin and kidney blood vessels [37,62].

Following a different approach, the administration of autoantibodies has been used to generate animal models. An example is the one induced by administration of human topoisomererase-1 autoantibodies with Freund´s adjuvant. In the same way, the transfer of anti-endothelin receptor type (ET_A_R) and anti-angiotensin receptor type-1 (AT_1_R) autoantibodies was associated with the development of obliterative vasculopathy [63].

### 3.5. Other Genetically Engineered Animal Models That Are Not Fully Characterized

There are other animal models of fibrosis including conditional peroxisome proliferator-activated receptor-γ (PPARγ)-deficient mice, relaxin knockout mouse, phosphatase and tensin homolog (PTEN) knockout mice, and those with overexpression of Wnt10b [38,63], which develop skin fibrosis. These models point to a relationship between these targets and fibrotic mechanisms. Other animal models have explored loss-of-function of different targets to evaluate their effect on fibrosis models induced by fibrotic agents (i.e., bleomycin). These include early growth response protein-1 (Erg-1)-deficient mice, macrophage chemoattractant protein-1 (MCP-1)-deficient mice, and microsomal prostaglandin E2 sythase-1 (mPGES-1)-deficient mice, in which a decrease in fibrosis has been observed [38,64]. All of these models have provided evidence on the mechanisms of fibrosis. However, it is not clear whether they present the three SSc hallmarks, namely, inflammation and autoimmunity, vasculopathy, and fibrosis, all of which participate in the complex pathophysiology of SSc. A description of the main animal models used for SSc can be found in Table 1.

**Table 1:** Representative animal models of systemic sclerosis. The most relevant characteristics of each model are highlighted.

Model	Target	Function	Mechanism	Fibrosis	Vasculopathy	Inflammation	Autoimmunity	Clinical Evidence	References
Bleomycin-mediated (various mice strains with different susceptibility, C3H/He and B10.A being the most susceptible	Non-specific target	Not applicable	TGF-β, collagen, and ECM synthesis upregulation EC and FB activation IL-4, IL-13, and CCL2 upregulation Oxidative stress and activation of NLRP3 inflammasome resulting in collagen synthesis STAT4 has also been involved	Dermal sclerosis Lung fibrosis	Vascular wall thickening (in deep dermis) has been reported	Present	Topo I, anti-U1 RNP, anti-histone autoantibodies Autoantibody that cross-reacts with gastric mucosa	Case reports of SSc-like syndrome in patients with antitumoral bleomycin	[22,32,37,38,39,63,64,65,66]
ROS-induced: Hydroxyl radicals or hypochlorous acid induced (Several strains) Peroxynitrites induced	Involvement of oxidized topo I and oxidative stress	Hydrogen peroxide production by endothelium, monocytes and fibroblasts	Involvement of AOPP, increased synthesis of collagen driven by ROS Activation of ADAM17/NOTCH, PDGFR, and VEGFRs is involved	Skin fibrosis Lung fibrosis Renal involvement	Renal vasculopathy	Present	Topo I	High serum levels of AOPP in dcSSc patients and lung fibrosis vs. controls	[33,34,38,40,63,64,67]
TGF-β and CTGF-induced	TGF-β and CTGF signaling	TGF-β, collagen type I, and CCL2 upregulation	TGF-β and CTGF play a role in inducing granulation tissue and fibrosis and increasing synthesis and remodelation of ECM proteins	Skin fibrosis	Not reported	Present	Not reported	See TβRΙΙΔk-fib	[32,43]
Angiotensin-II-induced	Angiotensin II receptor signaling activation	Upregulation of CTGF, TGF-β, and endothelial-to-mesenchymal transition MMP-12 has been related to fibrosis and vasculopathy	Administered angiotensin II induces vascular constriction, dermal fibrosis, and inflammation through activation of the TGF-β pathway. Increases fibrocytes and myofibroblasts in skin	Skin fibrosis	Present, including cardiovascular remodeling	Present	Not reported	High levels of Angiotensin II in serum of dcSSc patients. A subgroup of cases presented elevated angiotensin II signaling	[38,40,63,68]
Tsk1/^+^	Fibrillin-1 gene	Fibrillin is a structural protein of ECM. It has a direct interaction with latent TGF-β binding protein. It is thought that the failure of sequestration of the large latent complex drives an increase in the activation of TGFβ	Hyper-response to IL-4, MCP-3, and TGF-β with increasing synthesis of type I collagen B cell activation resulted from activation of CD19 signaling	Hypodermal fibrosis Emphysema-like disease with little fibrosis	Abnormal vascular tone and cardiomyopathy	Absent	Topo I and fibrillin-1 autoantibodies	Cases of human stiff skin syndrome associated with Fbn1 mutation Association of Fibrillin-1 SNP haplotypes with SSc in Choctaw Indians and Japanese populations	[32,37,38,40,69,70,71,72]
Tsk2/^+^	Col3α1	Collagen III synthesis	Gain-of-function mutation that results in increased synthesis of type I and III collagen	Dermal fibrosis	Absent	Present	Antinuclear, topo I/Scl70, anti-centromere, and anti-dsDNA antibodies		[22,32,38,41,73]
UCD-200 and 206 chickens	TGFBR1, IGFBP3, EXOC2/IRF4, CCR8, and SOCS1	Associated genes play a role in either SSc or autoimmunity	Spontaneously develops vascular damage, fibrosis and inflammation.	Skin fibrosis Lung fibrosis Internal organ fibrosis	Vasculopathy Intimal proliferation with narrowing of arterioles and capillaries and cell infiltration	Present	Polyarthritis Antinuclear antibodies, AECA, aCL antibodies, and rheumatoid factor	Not reported	[5,32,37,38,63]
Kinase-deficient TGFβRII model: TβRΙΙΔk-fib	Type II TGF-β receptor	Binding of the ligand to TβRII drives the phosphorylation of serine residues within the type I receptor (TβRI) to initiate TGF-downstream signaling	Paradoxical activation of TGF-β signaling It is postulated that accessory proteins and a nonsignaling type I receptor can modulate TβRII function	Skin fibrosis Pulmonary fibrosis Fibrotic cardiomyopathy	Generalized vascular remodeling	Not present	Not present	Several lines of research support the ling of link between TGF-β and fibrotic disease and systemic sclerosis	[22,32,38,39,40,43,74,75,76]
TBRI(CA); Cre-ER mice	Expression of a constitutionally active TGF-β1 type I receptor	TGF-β signaling activation	Increased collagen synthesis	Progressive and generalized dermal fibrosis Myocardial fibrosis Altered aortic dynamics	Thickening of blood vessel walls in small arteries of the lung and kidney Increased levels of von Willebrand factor Altered endothelin-1 signaling	Absent	Not reported	See TβRΙΙΔk-fib	[38,39,63,77]
Fbn-1 mutations Knock-in mouse: SSS- associated change W1572C (WC/WC, WC/+) D1545E (DE/+) (Homozygous was lethal)	FBN1 gene	See fibrillarin function in Tsk1 model	Excessive elastin, collagen, and microfibrillar aggregates in dermal TGF-β upregulation	Skin fibrosis	Not reported	Present	Topo I	See Tsk1 clinical evidence	[22,78]
Fra-2 Tg mice	Fra-2 overexpression	A member of the Fos family of transcription factors, a downstream mediator of TGF-β and PDGF. Induced by cellular stress and controls apoptosis, inflammation, healing and proliferation.	Upregulation of type I collagen in dermal fibroblasts, EC apoptosis and epithelial-to-mesenchymal transition, PDGF signaling activation	Skin fibrosis Pulmonary fibrosis	Vasculopathy Decrease in the number of capillaries EC apoptosis, pulmonary arterial occlusion	Present	Not present	Fra-2 expression is elevated in SSc dermal fibroblasts, EC, and pneumocyte epithelial cells	[38,40,63]
Fli1^ΔCTA/ΔCTA^	Fli1	Fli1 has roles in hematopoiesis and vasculogenesis, serves as a transcriptional repressor through its CTA domain, inhibits collagen genes, and is a negative regulator of ECM	Upregulation of dermal fibrillar collagen and Fli1 protein levels	Skin fibrosis	Present. Includes increases vascular permeability, similar to the one observed in SSc	Not reported	Not reported	Fli1 proteins are reduced in dermal fibroblasts, EC, and SMC of SSc patients	[18,38,40]
Fli1 ECKO Conditional deletion of Fli1 in EC	Fli1	Fli1 is a transcription factor expressed in EC and hematopoietic cells. It participates in the regulation of development and differentiation of EC and vasculature	Loss of endothelial integrity Fli-1 depletion has an effect on expression of genes that regulate vascular integrity	Absent	Present, irregular diameter and disorganization of the dermal vascular network	Absent	Not reported	See Fli1 mutated mice	[40,63]
Fli1-KLF5-KO	Fli1+KLF5	Fli1 is a potent repressor of type I collagen gene KLF5 has a potentially synergic effect with Fli1	Activation of both canonical and non-canonical TGF-β signaling Upregulation of ECM genes and CTGF	Skin fibrosis Lung fibrosis	Obliterative vasculopathy with vascular stenosis, loss and bushy skin capillaries Progressive obliteration of pulmonary arterioles	Present	Antinuclear antibodies	Downregulation of KLF5 in skin samples and SSc fibroblasts Fli1 repression in SSc fibroblast and EC	[20,22]
Sirt3-deficient mice	Sirt3	Class III histone deacetylases that play a key role in maintenance of mitochondrial integrity	Deficiency induces spontaneous multiorgan fibrosis as the mice age, accompanied by oxidative stress and mitochondrial damage	Multiorgan fibrosis including in the lungs and heart	PAH	Not reported	Not reported	Marked suppression of Sirt1 has been found in skin biopsies and explanted fibroblasts from SSc patients in two reports	[22,79]
Endothelin-1 Tg	ET-1 overexpression	Potent vasoconstrictor, regulation of blood flow in microvascular beds, sodium and water reabsorption Stimulates proliferation and collagen synthesis in normal fibroblast	Fibrogenic effect including: increased pulmonary matrix synthesis with chronic inflammation, excessive collagen production of SSc fibroblasts enhanced by TGF-β	Renal fibrosis Lung fibrosis (chronic overexpression)	Increased media/lumen ratio of intrarenal arteries	Present	Not reported	Higher serum levels and overproduction of endothelin-1 in SSc patients and in primary RP. Levels were associated with skin score and disease duration Effectiveness of endothelin-1 blockade in pulmonary hypertension and RP treatment	[38,48,49,80,81,82,83]
PDGFR-α hyperactivation	PDGFRα conditional overexpression	PDGFs play an important role in the development and maintenance of connective tissue, they are potent mitogens and chemoattractants of mesenchymal cells, they exert their biological effects through binding of PDGFRα/β	Activation of cellular programs that generate connective tissue Connective tissue hyperplasia and increased extracellular matrix deposition	Skin fibrosis Internal organ fibrosis	Not reported	Not reported	Not reported	Increased levels of PDGF and PDGFR in skin and lung samples of SSc patients Presence of stimulatory autoantibodies directed toward PDGFR More common and intense PDGFRβ inmunoreactivity in small vessels of SSc-associated PAH compared to idiopathic PAH	[51,84,85,86,87]
Cav-1−/− C57B1/6KO mice	Caveolin-1	It is an integral membrane protein and a structural component of caveolae. It modulates the activity of caveolae and disrupts TGF-β signaling	Upregulation of TGF-β and ECM proteins ROS, HIF1α y NFκB signaling pathways are activated	Thickening of alveolar septa with uncontrolled hyperproliferation of angioblastic cells and fibrosis Skin fibrosis	Vascular system disfunction Increased accumulation of collagen fibers around blood vessels of the deep dermis	Present	Not reported	Reduced levels of Cav-1 in skin and dermal fibroblasts from SSc patients and in lung samples from ILD-SSc patients Reduction of Cav-1 in lung fibroblasts and lung tissue in bleomycin- induced IPF	[52,53,54,56]
Hematopoietic cell transplantation: Scl-GVHD LP/J/ C57BL/6J B10.D2/BALB/c mice RAG-2 KO mice	Non-specific target	TGF-β upregulation, increased type I collage, chemokines, and other growth factors	Fibroblast, T cells, and other leucocytes involved	Skin fibrosis Lung fibrosis (not all models) Involvement of internal organs (kidney, small intestine) (not all models)	Vasculopathy Renal crisis	Present	Autoantibodies	Scl-GVHD after hematopoietic cell transplantation	[32,38,39,61,62,63,72,88]
Topo I and CFA-induced SSc	DNA topoisomerase	Relaxation of supercoiled DNA	Upregulation of TGF-β, IL-17 and IL-6	Skin fibrosis Pulmonary fibrosis	Not reported	Present	Topo I	Several studies reported the presence of autoantibodies including disease-specific autoantibodies	[38,40,63,89]

AECA: anti-endothelial cell autoantibodies, aCL: anticardiolipin antibodies, AOPP: advanced oxidation protein products, Cav-1: caveolin-1, CFA: Complete Freund´s Adjuvant, dcSSC: diffuse cutaneous systemic sclerosis, EC: endothelial cell apoptosis, ECM: extracellular matrix, ET-1: endothelin-1, DE/+: heterozygous D1545E mice, KO: knockout, HIF1α: hypoxia inducible factor 1α, PAH, ILD: interstitial lung disease, IPF: idiopathic pulmonary fibrosis, pulmonary arterial hypertension, MCP-3: monocyte-chemotactic protein-3, NFκB: nuclear factor κB, PDGF: platelet derived growth factor, PDGFR: platelet derived growth factor receptor, ROS: reactive oxygen species, RP: Raynaud´s phenomenon, Tg-mice: transgenic mice, TGF-β: transforming growth factor β, Topo I: anti-DNA topoisomerase I, SSc: systemic sclerosis, Sirt3: sirtuin 3, Scl-GVHD: sclerodermatous graft-versus-host-disease, SMC: smooth, muscle cell, SNP: single nucleotide polymorphism, VEGFR: vascular endothelial growth factors receptor, WC: W1572C mice, WC/WC: homozygous mice, WC/+: heterozygous mice.

Most models, as other authors have pointed out, have used rodents (mainly mice), given the homology to the human genome and other technical and genetically engineered advantages. However, to date, no animal model has been able to recreate proliferative obliterative vasculopathy of nailfold capillaries as observed in SSc patients, or at least it does not seem to have been previously evaluated.

### 3.6. Nailfold Capillaroscopy in the Guinea Pig

**Figure 1:**
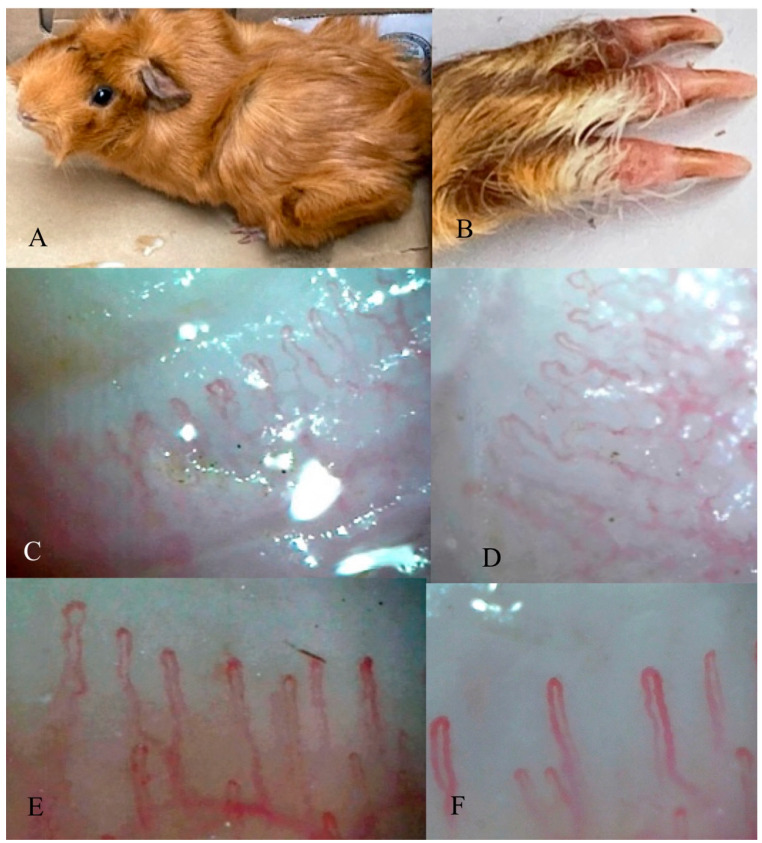
Human and animal NFC. (**A**) Guinea pig specimen used for NFC. (**B**) Guinea pig nailfold used for NFC. (**C**,**D**) Guinea pig NFC. (**E**,**F**) normal human NFC. NFC: nailfold capillaroscopy.

In order to design and propose an animal model that resembles RP (primary or secondary) with abnormal nailfold capillaroscopic findings such as the ones observed in SSc and other autoimmune diseases, it must first be elucidated whether animal nail fold capillaroscopy can be observed. For this purpose, the nailfold (*n* = 1) of the guinea pig (*Cavia pocellus)* was investigated by nailfold capillaroscopy (NFC). NFC was performed with a stereoscope VE-S7 with a magnification of 55× (Velab Co., Pharr, TX, USA) and a capillaroscope model JH-1004C with a total magnification of 380× to obtain images recorded with the software MicroCirculationXP (Jiansu Jiahua Electronic Instrument Co., Xuzhou, China). NFC was performed at room temperature on the front and hind limbs of the specimen. The excess hairs near the nailfold were trimmed, and immersion oil was applied to the nailfold. First, a panoramic view of the nailfold was carried out with the stereoscope at a magnification of 55×. Subsequently, the capillaries were observed at a total magnification of 380× with the capillaroscope. Figure 1 shows representative images of the capillaries of the nailfold of the right hind limb of the specimen at 380× (Figure 1C,D). The nailfold capillaries could be observed, and showed a very similar anatomical pattern to the human nailfold at 380× (Figure 1E,F). The number of capillaries and capillary flow could also be observed, and the diameters of the capillaries were measurable. No pharmacological or invasive procedures were performed prior to NFC in the guinea pig. The specimen was kindly lent by Dr. Ernesto López from a private animal care facility.

In normal NFC, the capillaries are usually homogeneously arranged and present a hairpin or “U” shape. They are distributed with their major axis parallel to the skin surface in a pattern described as a “comb-like structure”. However, due to the variability within a population, tortuous and crossed capillaries can be observed with high prevalence, and to a lesser degree, other shapes such as ectatic, bushy or bizarre loops can be observed. The structure and shape are best seen in the most distal row of capillaries. The normal density of capillaries is between 9 and 14 per mm, and the normal diameter of the afferent limbs ranges between 6 and 19 μm and 8 and 20 μm for efferent limbs. The average length of nailfold capillaries can be between 200 and 500 μm, but some of them may seem shorter. A large network of vessels away from the distal row of hairpin capillaries corresponds to the subpapillary venular plexus (SPVP) and is visible in a proportion of subjects more frequently in the 5th finger [90].

NFC parameters in the guinea pig were similar to the values for a healthy human NFC, with 13 capillaries per mm, an afferent limb diameter of 7 μm, efferent limb diameter of 9 μm, and apical limb diameter of 8 μm. The average length was 166 μm, and SPVP could also be observed. This pattern, in general, seems very similar to a human NFC. It is of note in the guinea pig NFC that just one line of hairpin-shaped capillaries seems to be observed distal to SPVP (Figure 1).

Clinically, the observation of capillaries by NFC has gained great relevance in several diseases such as Diabetes Mellitus and hypertension [91,92,93,94,95], but especially in autoimmune rheumatic diseases including diagnosis and as a potential prognostic tool for SSc [95,96,97]. Being a non-invasive, simple and relatively low-cost technique, its main use in clinical practice and in research has been with human subjects. However, its potential use in animal models for the research of the pathophysiology of microangiopathic disease has been missed.

Once we observed that NFC is indeed possible in the guinea pig, NFC in a larger number of wild type guinea pigs and murine models is planned to describe the anatomy of nailfold microstructure under “normal” conditions, as well as in known animal models of SSc for both fibrosis and vascular damage. The need for further validation of anatomical characteristics of this model with a larger sample should be noted, as well as the need to evaluate its application to previously described mice models of SSc.

Once the anatomical characteristics of the models have been described, the goal is to develop an RP model by targeting known mechanisms of RP pathophysiology of primary and secondary RP, such as endothelin-1 and vascular endothelial growth factor (VEGF), among others. The development of these models has, as can be inferred, application in other pathways involved in the disease. Additionally, they may allow development of novel therapies for the disease, especially for vascular features. Although at present there is an vast therapeutic arsenal for vascular manifestations, it does not cover all needs, can be ineffective, and has a high rate of therapeutic failure [3]. In previous work, the possible relationship between capillary abnormalities and leukotrienes was shown. New studies are required to investigate and corroborate this relationship. Animal models that use exogenous agents and gain-or-loss-of-function transgenesis targeting leukotriene pathways and its interaction with other mediators are critical to study the pathophysiology of microangiopathy in greater depth. There is great interest by several study groups, including the authors, in the role of leukotrienes in the pathophysiology of SSc because of its biological activity, which corresponds to the pathogenic triad of SSc [98,99,100,101]. Assessment of microangiopathy in NFC of SSc animal models or RP models could enable research of leukotriene pathways, their interactions with other mediators (i.e., TGF-β, Hypoxia inducible factor 1α, among others), and the usefulness of antileukotriene therapy and other pathogenic mediators or potential antifibrotic therapies in SSc.

## 4. Conclusions

Currently, there are no animal models that simultaneously present all three main pathological mechanisms of SSc or models that recreate RP. The potential use of NFC to evaluate microcirculation in current models has not been explored. We showed that NFC can be observed in the nailfold of the guinea pig, but could be also applicable to murine models, and is morphologically very similar to human NFC. This encourages the development of RP animal models that could be used for the study of microvascular diseases, including SSc, other autoimmune, and chronic diseases.

## Data Availability

Not applicable.
